# In Situ Control of Cell Substrate Microtopographies Using Photolabile Hydrogels

**DOI:** 10.1002/smll.201201841

**Published:** 2012-10-17

**Authors:** Chelsea M Kirschner, Kristi S Anseth

**Affiliations:** Department of Chemical and Biological EngineeringUniversity of Colorado, 596 UCB, Boulder, Colorado, 80303-1904, USA; The Biofrontiers Institute, University of Colorado596 UCB, Boulder, Colorado, 80303-1904, USA; The Howard Hughes Medical Institute, University of Colorado596 UCB, Boulder, Colorado, 80303-1904, USA

**Keywords:** hydrogels, biomaterials, microstructures, photolysis, cell adhesion

## Abstract

Substratum topography can play a significant role in regulating cellular function and fate. To study cellular responses to biophysical cues, researchers have developed dynamic methods for controlling cell morphology; however, many of these platforms are limited to one transition between two predefined substratum topographies. To afford the user additional control over the presentation of microtopographic cues to cell populations, a photolabile, PEG-based hydrogel system is presented in which precisely engineered topographic cues can be formed in situ by controlled erosion. Here, the ability to produce precisely engineered static microtopographies in the hydrogel surface is first established. Human mesenchymal stem cell (hMSC) response to topographies with features of subcellular dimensions (∼5 to 40 μm) and with various aspect ratios increasing from 1:1 to infinity (e.g., channels) are quantified, and the dynamic nature of the culture system is demonstrated by sequentially presenting a series of topographies through in situ modifications and quantifying reversible changes in cell morphology in response to substratum topographies altered in real time.

## 1. Introduction

Adult stem cells such as human mesenchymal stem cells (hMSCs) are multipotent cells that contribute to tissue regeneration.^1^ In response to a trigger such as trauma or disease, hMSCs circulate away from their niche and engraft in mechanically diverse environments to renew bone, cartilage, muscle or fat.^2^ The extracellular matrix (ECM) in these diverse microenvironments is complex and presents cells with both nano- and micro-topographies, along with a complex milieu of biochemical cues. Static topographic cues have been shown in vitro to influence cell adhesion,^3^ migration,^4^ proliferation,^5^ gene expression^5^ and protein expression.^6^ Cell shape, cell spreading and cytoskeletal tension, independent of soluble factors, have also been demonstrated to have a strong influence on the lineage commitment of hMSCs.^7^,^8^

To better understand how extrinsic cues trigger mechano-sensitive pathways, which subsequently convert these biophysical cues to biochemical signals that specify lineage commitment,^9^ several different material platforms have been exploited to dictate cell shape and spreading. Microcontact printing of the ECM protein fibronectin (Fn) was used to show that hMSCs became osteoblastic when allowed to adhere, flatten and spread, while unspread, rounded cells underwent adipogenesis.^7^ Kilian et al. recently corroborated these results using stamped shapes of self-assembled monolayers that promoted adsorption of Fn. These studies also demonstrated that the aspect ratio and curvature of geometric features also influence hMSC lineage commitment (e.g., features that increased acto-myosin contractility promoted osteogenesis).^8^ Pre-printed nanotopographies replicated in polycaprolactone (PCL) through hot embossing retained hMSC multipotency for eight weeks in culture when presented in an ordered arrangement and promoted osteogenic differentiation when the same features were presented in a disordered array.^10^ Trappmann et al. recently elucidated a mechanism by which cells respond to various material interfaces by demonstrating that the interface between the cell and the ECM is different on materials of different stiffnesses. Characterization of these interfaces revealed pore sizes ranging from the nano- to microscale and resulted in surface topographies that caused variation in anchoring distances between covalently bound ECM proteins. It was demonstrated that cells exert a mechanical force on the ECM, and the mechanical feedback from this protein interface, which could be influenced by the underlying substrate topography, mediated cell fate.^11^ Collectively, this seminal body of work has led to a nascent understanding of how cell shape and spreading influence lineage commitment; however, static cues (e.g., pre-printed topographies) fail to reproduce the dynamic nature of the ECM.

In order to investigate how mechanical cues influence cell biology, culture platforms with biophysical cues that are tunable in situ are currently being developed using dynamically responsive moieties. Recently, dynamic control of cell morphology has been achieved through exploiting the unique properties of shape-memory polymers, which change shape upon exposure to a stimulus such as temperature. For instance, Henderson and colleagues embossed reversible microgrooves into NOA-63, a polyurethane-based thiol-ene cross-linked polymer system.^12^ Transitions between the micro-grooved surface and the original smooth surface controlled fibroblast alignment and cytoskeletal organization.^12^ Le et al. developed a dynamic cell culture substrate by first cross-linking a PCL-based shape-memory polymer in a mold to produce an initial shape and then mechanically deforming the primary shape to form a secondary shape at a temperature above the transition temperature of the system.^13^ These substrates result in the ability to transition between two pre-defined shapes, instead of simply changing from a smooth substrate to a topographically modified one to control hMSC morphology.^13^ One disadvantage to these stimuli-responsive topographies is that the shape-memory polymer systems are limited to two transitions, one from the original shape to the secondary shape and another back to the original. In this way, it is only possible to study cellular responses to two topographies at a time.

To provide a complementary approach with the potential to overcome this limitation, we developed a cell culture substrate based on a photodegradable poly(ethylene glycol) (PEG) hydrogel platform recently developed in the Anseth laboratory^14^ that is compatible with cell culture conditions.^15^,^16^ Photodegradable hydrogels have been used to create cell culture platforms that present dynamic biochemical^17^,^18^ and biophysical cues such as matrix stiffness^16^,^19^ to cell populations. Photolysis of these hydrogels has also been employed to deliver soluble factors with spatial and temporal control.^20^ In this contribution, the photolabile linkages in the hydrogel system are exploited to allow for creation of topographies using precise spatial erosion, while subsequently allowing the user to dynamically present a series of precisely engineered topographic cues to a cell population in real time. Specifically, we use photolithographic techniques to pattern hydrogels and characterize erosion depth and feature size. We also seed hMSCs onto the patterned and unpatterned photolabile PEG hydrogels, to monitor and quantify cellular morphology in response to sequentially presented subcellular microtopographies during cell culture. The results demonstrate control of the average cellular aspect ratio and cellular orientation through the presentation of a series of microtopographic cues. This dynamically tunable cell culture system may serve as a powerful tool to improve our understanding of how hMSCs respond to real time changes in physical cues in their microenvironment.

## 2. Results and Discussion

### 2.1. Photodegradable Hydrogel Preparation and Topographic Modification

**Figure 1:**
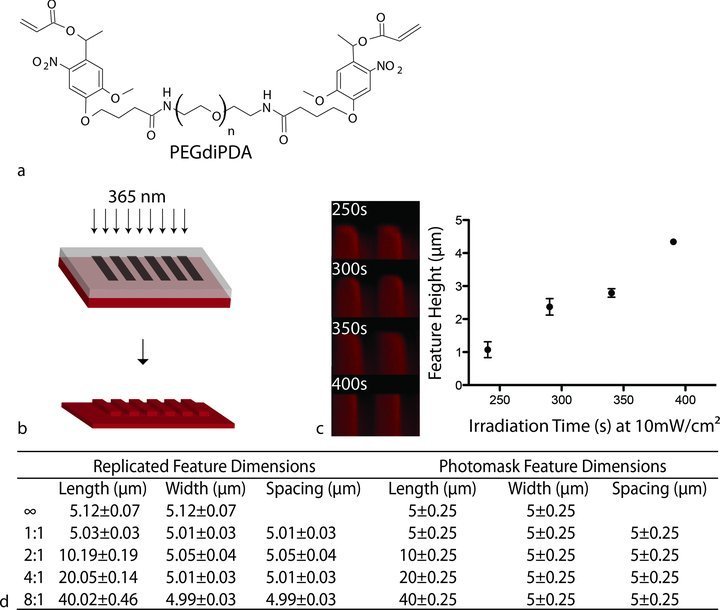
Fabrication and characterization of topographically modified hydrogel substrates. a) The photolabile hydrogels were synthesized via a redox-initiated free radical chain polymerization of PEGdiPDA with PEGA. b) Schematic of the photolithography process. A chrome on quartz photomask was placed in close contact with the photolabile hydrogel surfaces, which were exposed to 365 nm, collimated light at 10 mW/cm^2^. c) Feature depths represented by z-stacks from confocal microscopy and profilometry measurements of photolabile hydrogels at different irradiation times (365 nm, 10 mW/cm^2^). Feature depth increases with irradiation time at a fixed wavelength and intensity. Error bars, 95% confidence intervals. d) Measurement of topographic features in the x-y plane (mean ± standard deviation) shows high fidelity of pattern transfer compared to the features defined by the photomask.

Photodegradable hydrogels were synthesized through a redox-initiated free radical chain co-polymerization of a PEG diacrylate cross-linking macromer (PEGdiPDA) (Figure 1a) that was synthesized by coupling a nitrobenzyl-based photodegradable acrylate (PDA) to PEG-bis-amine^21^ and PEG monoacrylate (PEGA). The gel forming monomer solution contained 8.2 wt% PEGdiPDA (M_n_≈ 4070 g mol^−1^), 6.8 wt% PEGA (M_n_≈ 400 g mol^−1^), 300 nM human Fn and 0.2 M ammonium persulfate in phosphate buffered saline (PBS, pH = 7.4). Due to the bioinert nature of PEG-based hydrogels,^22^ Fn was entrapped in the network to promote cell adhesion.^23^ The polymerization was initiated by adding 0.1 M tetraethylmethylenediamine (TEMED). Hydrogel films (thickness 0.25 mm) were covalently linked to glass coverslips (diameter 18 mm) during polymerization with a silane-coupling agent.

To create topographically modified surfaces, hydrogels were degraded by controlled irradiation with 365 nm collimated light at 10 mW/cm^2^ through a chrome on quartz photomask (Figure 1b) resulting in gel erosion and feature heights that scaled with UV exposure time as measured by contact profilometry (Figure 1c). For cell culture experiments, an exposure time of 250s was selected to fabricate features ∼1 μm in depth to allow for cell spreading across the surface without the physical constraint that would be caused by deeper features.^24^ This approach also enables the guidance of a larger cell population than microcontact-printed features for single cell manipulation. Another advantage of the photodegradable hydrogel system is that the hydrogels can be swollen to equilibrium prior to photolithographic patterning. In this way, the final feature sizes produced in the material are close to those originally defined by the photomask. The photolabile hydrogels were covalently and fluorescently labelled with methacrylated rhodamine and imaged with confocal microscopy. Feature dimensions were measured using ImageJ software, and the average final dimensions were all within 1% of the originally defined feature size demonstrating high fidelity of pattern transfer (Figure 1d).

**Figure 2:**
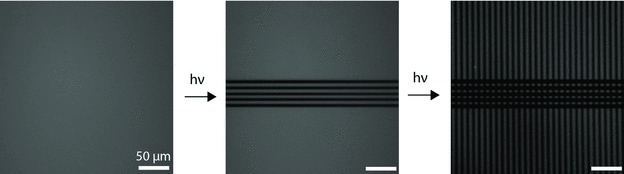
Fluorescently labeled, photodegradable PEG-based hydrogels demonstrate the ability to introduce unique topographies spatially and temporally. The smooth surface was patterned into channels and subsequently into squares using two-photon photolysis using an LSM 710 (Zeiss) with a mode-locked Ti:Sapphire, femtosecond pulsed, multiphoton laser (Chameleon Ultra II, Coherent, Inc.) at 740 nm. Two-photon photolysis affords the ability to create interface multiple unique topographies on the same sample.

Importantly, photolysis of PEGdiPDA is achieved using cytocompatible irradiation conditions. Previous studies have also demonstrated that the degradation products of this hydrogel formulation are biocompatible, and no adverse effects on cell viability were observed during these experiments.^14^,^16^,^25^ This patterning process can be repeated to alter the surface topography in the presence of cells by subsequent single photon irradiation (365 nm) through a photomask or by two-photon photolysis at 740 nm.^25^ Figure 2 depicts an example of a series of patterning steps; a smooth gel was patterned into an anisotropic channels topography and subsequently into an isotropic squares pattern using two-photon irradiation. These photo-controlled manipulations of surface topography demonstrate some key advantages over recently developed technologies (e.g., thermoresponsive, shape-memory polymers).^12^,^13^ The first advantage is the ability to maintain cell cultures under standard conditions (37 °C with 5% CO_2_) while altering the surface and the ability to perform multiple, sequential alterations that are not predetermined by the substrate material. It is also possible to create multiple topographies spatially or alter only part of a pattern to produce topographic interfaces within a single sample (Figure 2). The alterations are spatially and temporally user-controlled, which can be leveraged to probe the effects of microtopographic cues in powerful new ways.

### 2.2. Cellular Responses to Static Microtopographies

**Figure 3:**
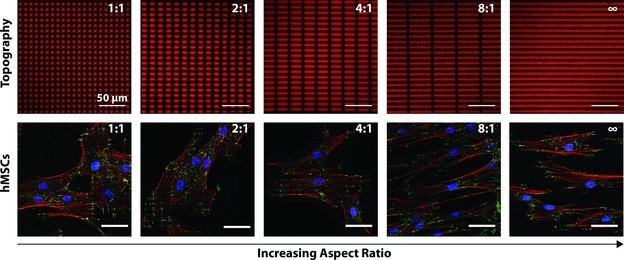
hMSCs exhibited contact guidance on substrate topographies. Topographies were produced by photolithography (365 nm, 10 mW/cm^2^, 250s) resulting in feature depths of ∼1 μm. Cytoskeletal components including focal adhesions (green, anti-vinculin), F-actin (red, phalloidin), and cell nuclei (blue, DAPI) are stained here after 3 days in culture to demonstrate that topographic features influenced cellular elongation, alignment and focal adhesion placement. Cellular elongation and alignment increased (qualitatively here) with increased feature aspect ratio.

**Figure 4:**
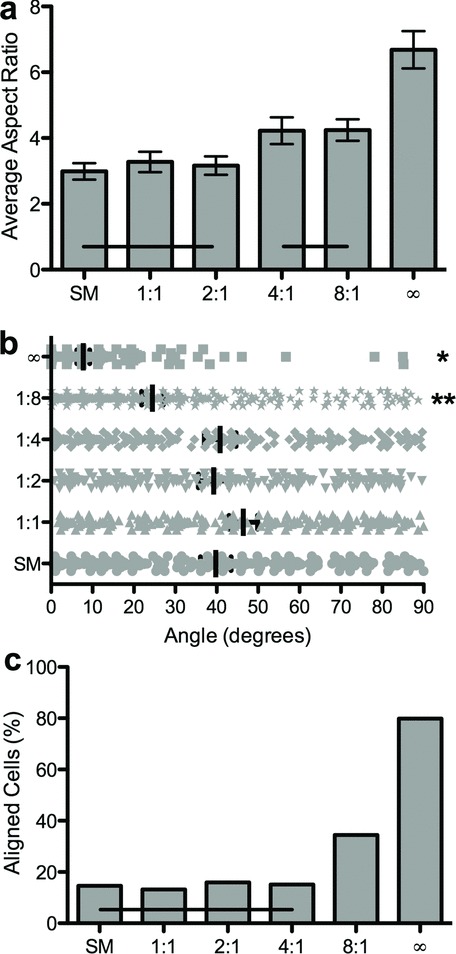
Quantification of hMSC morphology on static microtopographies. a) Average cellular aspect ratio is plotted versus topographic feature aspect ratio. Horizontal bars indicate no statistical difference (ANOVA, Tukey Test, α = 0.05). b) The distribution of cellular orientation angles on each substrate. Stars indicate statistically different distributions (ANOVA, Tukey Test, α = 0.05). c) The percentage of aligned cells is plotted versus the topographic feature aspect ratio. Horizontal bars indicate no statistical difference (Marascuilo procedure, α = 0.05). Quantitative increases in elongation and alignment were measured on patterns with increasing aspect ratios of features.

To demonstrate the utility of our photodegradable hydrogel platform, hMSCs were seeded onto topographies with features of subcellular dimensions (∼5 to 40 μm) with various aspect ratios increasing from 1:1 to infinity (e.g., channels). The cells were cultured for 3 days in growth media and then fixed and stained for cytoskeletal components including F-actin and vinculin (Figure 3). Qualitatively, these images show that hMSC elongation and orientation along the features increased with increasing aspect ratio and that surface topographies influenced focal adhesion placement. To track and measure cell morphology quantitatively, hMSCs were labeled with a fluorescent membrane dye (CellTracker Green CMFDA (5-Chloromethylfluorescein Diacetate), Invitrogen). Image analysis showed that the cellular aspect ratio increased as the aspect ratio of the topographic features increased (Figure 4a). Features with aspect ratios of 4:1 and 8:1 increased cellular aspect ratios to 4.2 compared to those measured on smooth, 1:1 and 2:1, which ranged from 3.0 to 3.2. The channels topographies produced the largest average cellular aspect ratio, 6.7 (ANOVA, Tukey Test, α = 0.05). Cellular orientation along the topographic feature axis showed a similar trend (Figure 4b). Both the 8:1 and channels topographies demonstrated significant increases in the percentage of cells aligned within 10 degrees of the topographic feature direction (Figure 4c) (Marascuilo procedure α = 0.05).

These static experiments demonstrate control over cell morphology with subcellular microtopographies, and that as the feature aspect ratios increase the average cellular aspect ratios and alignment along topographic features on the surface also increase. Single cell adhesion experiments have shown that cellular anisotropy influences hMSC lineage commitment.^8^,^26^ For example, osteogenesis was increased in cells with aspect ratios of 4:^8^,^26^ and 8:^26^ compared to isotropic surfaces. The results indicate that these average cellular aspect ratios are achievable with our photolabile platform. For this reason, the cell culture system can be exploited to study how cellular anisotropy influences hMSC differentiation.

### 2.3. Cellular Responses to Dynamic Microtopographies

**Figure 5:**
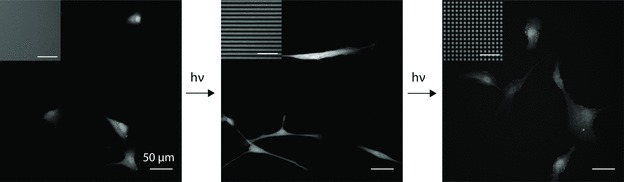
Representative images of hMSC morphology on sequentially presented dynamic microtopographies. hMSCs were initially seeded onto smooth surfaces, which were in turn patterned sequentially using photolithography (365 nm, 10 mW/cm^2^, 250 s) into an anisotropic (∞:1) channels pattern and then an isotropic (1:1) squares pattern. hMSCs were labeled with CellTracker Green CMFDA (5-Chloromethylfluorescein Diacetate), Invitrogen to track cellular morphology. Cells exhibited a more rounded morphology on the smooth pattern, elongated along the feature axis on the channels pattern and returned to a more rounded morphology upon exposure to the isotropic squares pattern.

**Figure 6:**
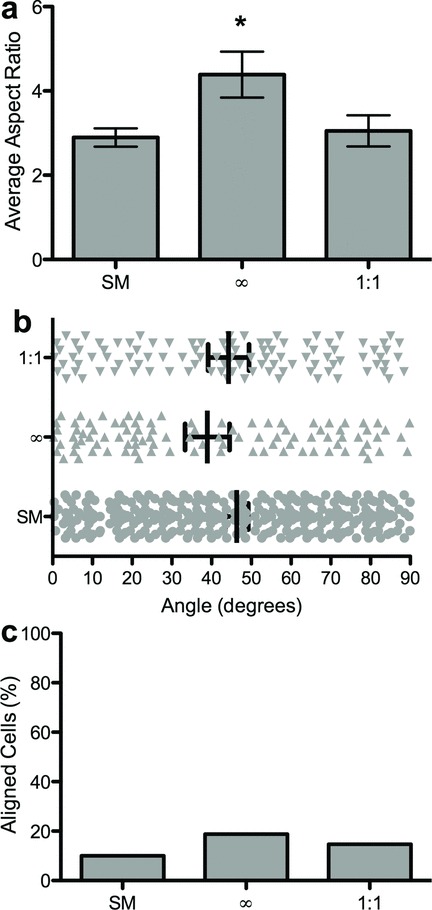
Quantification of hMSC morphology on dynamically presented microtopographies. a) Average cellular aspect ratio is plotted versus topographic feature aspect ratio. Stars indicate statistically differences (ANOVA, Tukey Test α = 0.05). b) The distribution of cellular orientation angles on each substrate. (no statistical differences, ANOVA, Tukey Test, α = 0.05). c) The percentage of aligned cells is plotted versus the topographic feature aspect ratio (no statistical differences, Marascuilo procedure, α = 0.05). Cellular elongation increased upon in situ exposure to the channels pattern and decreased to a more rounded morphology upon exposure to the isotropic squares pattern. Alignment followed a similar trend.

The photodegradable hydrogel platform was exploited to expose one cell population to a series of different surface topographies. hMSCs were initially seeded onto smooth surfaces, which were in turn patterned sequentially using photolithography (365 nm, 10 mW/cm^2^, 250 s) (Figure 5). Live cell imaging 24 h after patterning revealed that cell morphology and alignment had begun to respond and change based on the new underlying pattern. The presentation of channels (∞:1), an anisotropic pattern, led to a statistically significant increase in the average cellular aspect ratio (ANOVA, Tukey Test, α = 0.05) (Figure 6a) and an increase (not significant, Marascuilo procedure α = 0.05) in cellular alignment along the pattern (Figure 6b,c). These changes in cell morphology were reversed with the next patterning step, which returned the cells to an isotropic surface (e.g., squares (1:1)) (Figure 6). In this way, it will be possible to investigate the influence of dynamic changes on cellular anisotropy on hMSC lineage. For instance, temporal changes in topographic cues could trigger or preprogram cells to commit to a certain lineage.

Here, we sought to fabricate a stimuli-responsive cell-material interface that could be exploited to improve our understanding of how stem cells sense and respond to topographic cues in their microenvironment. Cellular focal adhesions are mechanosensitive, signalling complexes that have been shown to grow and adapt in response to cytoskeletal tension induced by topographically modified substrates.^27^ It is likely that topographic features alter the interaction of focal adhesions with a material interface, resulting in intracellular tension, which is an important regulator of cell fate.^7^,^8^,^10^ Previous work has shown that as the aspect ratio of single cells is increased, osteogensis also increases.^8^ Photo-induced, in situ manipulation of surface topography is a tool that can be used to study how mechanosensitive pathways are activated in cells and how this activation influences cell fate when changes in cell tension are induced. This knowledge could be exploited to direct stem cell function in vitro, i.e., expanding and differentiating autologous hMSCs for transplantation in cell therapy treatments or triggering differentiation on-demand in real time.

## 3. Conclusion

A photolabile-hydrogel cell culture platform is presented that allows users to control cell morphology in real time through the presentation of a series of precisely engineered microtopographies. Photolithographic techniques were employed to present both static and dynamic topographies to hMSCs in culture. Measurement of feature dimensions performed using image analysis and profilometry showed that final feature dimensions were within 1% of those defined by the photomask and that feature depth was controlled by time of exposure to UV irradiation. hMSCs exhibited contact guidance on all topographies. Cell morphology was quantified with image analysis. The most anisotropic patterns (e.g., 8:1 and channels) resulted in the highest average cellular aspect ratios and the most cellular alignment under static conditions. The presentation of sequential topographies resulted in measurable, reversible changes in cell morphology and alignment. This cell culture platform affords new opportunities for unprecedented, user-defined control of topographic cues in the presence of cells that can be exploited to improve our understanding of how cells, including progenitor and stem cells, respond to real time changes in physical cues in their microenvironment.

## 4. Experimental Section

*Synthesis of Photodegradable Cross-Linker*: A nitrobenzylether-based photodegradable monomer was synthesized by acrylation of ethyl 4-(4-(1-hydroxyethyl)-2-methoxy-5-nitrophenoxy)butanoic acid as previously described.^14^,^21^ Subsequent purification yielded a small molecule monomer that is photolabile and can be coupled to molecules containing primary amines through a pendant carboxylic acid. The photodegradable cross-linking macromer, PEGdiPDA, was synthesized by coupling the PDA to PEG-bis-amine (M_n_∼3,400 g mol^−1^, Laysan Bio).^21^ Briefly, PDA (5.9 mmol) was dissolved in *N*-methy-2-pyrrolidone (NMP, 104 mmol, Applied Biosystems) in a glass scintillation vial. The activation reagents, 2-(1H-Benzotriazole-t-yl)-1,1,3,3-tetramethylaminium hexafluorophosphate (HBTU, 6.5 mmol, Anaspec) and 1-hydroxyl-benzotriazole (HOBt, 6.5 mmol, Anaspec) were added, and the solution was vortexed and heated to begin dissolution. To activate the carboxylic acid, *N-N*-Diisopropylethylamine (DIEA,11.8 mmol, Anaspec) was added to the solution and stirred (5 min). Next, PEG-bis-amine (0.73 mmol) was dissolved in NMP (156 mmol) in a single-neck round-bottomed flask purged with argon with a magnetic stir bar. The activated acid solution was subsequently added to the PEG-bis-amine solution and the flask was purged with argon. The reaction was stirred overnight at room temperature. The reaction solution was then precipitated in ice-cold ethyl ether (Fisher Scientific). The precipitated product was recovered by centrifugation (5 min, 4400 rpm). The ether was decanted and the precipitated product was washed twice with ice-cold ethyl ether by repeated centrifugation. Following the final wash, the ether was decanted and the product was dried overnight under vacuum to remove any remaining ether. The product was dissolved in deionized water (DI water, 18 MΩ cm, Barnstead NANOpure II) and centrifuged (5 min, 4400 rpm) to remove precipitated HBTU/HOBt by-products. The remaining solution was decanted, dialyzed (SpectraPor 7, CO 1000 g mol^−1^) against DI water for 3 d at room temperature and lyophilized. The purity of the PEGdiPDA cross-linker was verified with proton NMR. ^1^H NMR (300 MHz, DMSO-_d6_,δ): 8.0 (t, 1H, C(=O)N*H*CH_2_CH_2_O), 7.6 (s, 1H, Ar-H), 7.2 (s, 1H, Ar-H), 6.4 (d, 1H, OC(=O)CH=C*H*_2_), d. 6.35 (m, 1H, AR-C*H*(CH_3_)OC(=O)CH=CH_2_), 6.25 (m, 1H, OC(=O)C*H*CH_2_), 6.05 (d, 1H OC(=O)CH=C*H*_2_), 4.1 (t, 2H, Ar-OC*H*_2_CH_2_CH_2_CO_2_H), 3.9 (s, 3H, Ar-OC*H*_3_), 3.5 (m, ∼304H, [CH_2_CH_2_O]_n_), 2.75 (t, 2H, N*H*_2_CH_2_CH_2_O), 2.4 (t, 2H, Ar-OCH_2_CH_2_C*H*_2_CO_2_H), 2.0 (m, 2H, Ar-OCH_2_C*H*_2_CH_2_CO_2_H), and 1.4 (d, 3H, Ar-CHC*H*_3_)

*Fabrication of Topographically Modified Hydrogels*: Photolabile hydrogels were synthesized through a previously described redox-initiated free radical chain copolymerization between PEGdiPDA and PEGA.^15^ The gel forming monomer solution contained 8.2 wt% (PEGdiPDA), 6.8 wt% PEGA (M_n_≈ 400 g mol^−1^, Monomer-Polymer and Dajac Labs) and 0.2 M ammonium persulfate (Acros) in phosphate buffered saline (PBS, pH = 7.4, Invitrogen). All components were sterile filtered to avoid contamination in cell culture conditions. For cell culture experiments Fn was entrapped in the network to promote cell adhesion by adding human Fn (300 nM in PBS, BD Biosciences) to the monomer solution.^23^ To visual gels fluorescently, methacryloxyethyl thiocarbamoyl rhodamine B (300 μm, Polysciences, Inc.) was added to the monomer solution. Monomer solutions were aliquoted into individual tubes for each sample. The polymerization was initiated by adding tetraethylmethylenediamine (TEMED, 0.1 M in PBS, Sigma Aldrich) to an aliquot of monomer solution while vortexing. This solution was then quickly pipetted into a mold made of two glass coverslips. One coverslip was siliconized with Sigmacote (Sigma Aldrich) to facilitate removal from the mold. The other coverslip was functionalized with an acrylate silane-coupling agent to covalently link the hydrogel to the coverslip. Coverslips (18 mm, No. 2, Fisher Scientific) were silanated as previously described^28^ via liquid deposition (0.5% 3-acryloxypropyl)-trimethoxysilane (Gelest) in 95% ethanol/water solution for 3 min and dried at 80°C. Photodegradable hydrogel samples were stored in a 12-well plate and swollen in PBS for at least 24 h before topographic modification.

To topographically modified surfaces, hydrogels were degraded by controlled irradiation by 365 nm light at 10 mW/cm^2^ through a chrome on quartz photomask or by controlled two-photon induced photolysis. Two-photon photoerosion was performed on an LSM 710 (Zeiss) with a mode-locked Ti:Sapphire, femtosecond pulsed, multiphoton laser (Chameleon Ultra II, Coherent, Inc.) at 740 nm with a 20x water immersion objective (NA = 1.0, Plan-Apochromat). Topographies were eroded by rastering the focal point through regions defined by the ZEN region of interest (ROI) software in the x-y plane while scanning in the z-direction. Photolithography was used to fabricate topographies for cell culture experiments and for subsequent patterning in the presence of cells. Swollen, smooth hydrogel samples were placed in direct contact with the chrome side of the photomask and exposed to 365 nm light at 10 mW/cm^2^ in a sterile biosafety cabinet for static experiments. After seeding cells onto hydrogel samples, photolithography was performed through a sterile mold consisting of a gasket (thickness 20 to 50 μm, Artus Corporation) and a coverslip (thickness No. 1, Fisher Scientific).

*Hydrogel Characterization:* Fluorescent z-stack images were taken on the LSM 710 of rhodamine-labeled hydrogels after topographic modification to measure feature dimensions and feature heights. ImageJ software was used to measure the dimensions of several features on each of three replicate samples in the x-y plane. Feature heights were visualized by compiling z-stack images and height measurements were confirmed by profilometry (DekTak 6M Stylus Profiler).

*hMSC Culture*: hMSCs were isolated from bone marrow (Lonza) and cultured in growth media (low-glucose DMEM, 10% fetal bovine serum, 1 ng/mL human fibroblast growth factor, 1 mg/mL amphotericin B, 50 U/mL penicillin and 50 mg/mL streptomycin, (Invitrogen)) under standard cell culture conditions (37°C with 5% CO_2_). Cells were passaged at ∼80% confluency and seeded on experimental samples at passage 3 in this study at 6000 cells cm^−2^. Immunostaining for cytoskeletal components was performed to visualize cell morphology. Cells were allowed to adhere and spread on test samples for 72 h. The cells were then fixed with 4% paraformaldehyde for 10 min and blocked with 5% bovine serum albumin (BSA, Sigma Aldrich) in PBS for 1 h. All immunostaining steps were performed at room temperature. Cells were incubated with monoclonal anti-vinculin antibody produced in mouse (1:400, Sigma Aldrich) in 3% BSA and 0.1% Tween 20 in PBS for 1 h. After rinsing, the cells were subsequently incubated with goat-anti-mouse IgG Alexa Fluor-488 (1:200, Sigma Aldrich) and Phalloidin-TRITC (1:200, Sigma) for 1 h and costained with DAPI (1:10,000, Sigma) for 15 min. Samples were mounted using Prolong Gold antifade reagent (Life Technologies) to preserve for imaging.

To monitor cell morphology, hMSCs were labeled with a fluorescent membrane dye (CellTracker Green CMFDA (5-Chloromethylfluorescein Diacetate), Invitrogen). A 10 mM stock solution of CellTracker Green CMFDA was diluted to a working concentration of 18 μM in serum-free medium and warmed to 37 °C. hMSCs were subsequently incubated with this solution for 45 min and then the medium was replaced with growth media for 30 min before seeding onto test samples. To quantify cell morphology cells were fixed after each experiment with 4% paraformaldehyde and mounted onto glass slides.

*Static and Dynamic Microscopy*: All static microscopy, i.e., imaging of fixed, mounted samples was performed on an LSM 710 (Zeiss). Dynamic, real-time live cell imaging was also performed on an LSM 710 (Zeiss). Briefly, CellTracker-labled hMSCs were seeded onto smooth hydrogel surfaces and cultured for 3 d in growth media. At this time point, cell-laden samples were loaded into a sterile mold consisting of a gasket (thickness 20 to 50 μm, Artus Corporation) and a coverslip (thickness No. 1, Fisher Scientific). The surfaces were imaged and then using photolithography patterned to create an anisotropic topography (e.g., channels). The freshly patterned surface was replaced in the incubator for 24 h after which it was reimaged and patterned a second time to create an isotropic pattern of squares. After 24 h the surface as imaged again.

*Image Analysis*: A MATLAB program was written to identify the CellTracker Green-labeled cells. The software approximated each cell as an ellipse and measured the long and short axes and orientation of each ellipse relative to the direction of the topographic features.

*Statistical Analysis*: All data are represented as mean ± 95% confidence interval. Statistical analysis including analysis of variance with Tukey Tests for multiple comparisons and plotting was performed using GraphPad software. The Marascuilo procedure was performed to identify statistical differences among proportions.
